# Serp-2, a virus-derived apoptosis and inflammasome inhibitor, attenuates liver ischemia-reperfusion injury in mice

**DOI:** 10.1186/s12950-019-0215-1

**Published:** 2019-05-29

**Authors:** Jordan R. Yaron, Hao Chen, Sriram Ambadapadi, Liqiang Zhang, Amanda M. Tafoya, Barbara H. Munk, Dara N. Wakefield, Jorge Fuentes, Bruno J. Marques, Krishna Harripersaud, Mee Yong Bartee, Jennifer A. Davids, Donghang Zheng, Kenneth Rand, Lisa Dixon, Richard W. Moyer, William L. Clapp, Alexandra R. Lucas

**Affiliations:** 1 Center for Personalized Diagnostics and Center for Immunotherapy, Vaccines and Virotherapy, Biodesign Institute, Arizona State University, Tempe, AZ USA; 2 The Department of Tumor Surgery, Second Hospital of Lanzhou University and The Key Laboratory of the Digestive System Tumors of Gansu Province, Lanzhou, China; 3 Department of Pathology, University of Florida, Gainesville, FL USA; 4 Divisions of Cardiovascular Medicine and Rheumatology, Department of Medicine, University of Florida, Gainesville, FL USA; 5 Department of Molecular Genetics and Microbiology, University of Florida, Gainesville, FL USA

**Keywords:** Ischemia-reperfusion injury, Liver, Serpin, Immune modulation, Inflammation, Necrosis

## Abstract

**Background:**

Ischemia-reperfusion injury (IRI) is an antigen-independent, innate immune response to arterial occlusion and ischemia with subsequent paradoxical exacerbation after reperfusion. IRI remains a critical problem after vessel occlusion and infarction or during harvest and surgery in transplants. After transplant, liver IRI (LIRI) contributes to increased acute and chronic rejection and graft loss. Tissue loss during LIRI has been attributed to local macrophage activation and invasion with excessive inflammation together with hepatocyte apoptosis and necrosis. Inflammatory and apoptotic signaling are key targets for reducing post-ischemic liver injury.

Myxomavirus is a rabbit-specific leporipoxvirus that encodes a suite of immune suppressing proteins, often with extensive function in other mammalian species. Serp-2 is a cross-class *ser*ine *p*rotease *in*hibitor (*serpin*) which inhibits the inflammasome effector protease caspase-1 as well as the apoptotic proteases granzyme B and caspases 8 and 10. In prior work, Serp-2 reduced inflammatory cell invasion after angioplasty injury and after aortic transplantation in rodents. In this report, we explore the potential for therapeutic treatment with Serp-2 in a mouse model of LIRI.

**Methods:**

Wildtype (C57BL/6 J) mice were subjected to warm, partial (70%) hepatic ischemia for 90 min followed by treatment with saline or Serp-2 or M-T7, 100 ng/g/day given by intraperitoneal injection on alternate days for 5 days. M-T7 is a Myxomavirus-derived inhibitor of chemokine-GAG interactions and was used in this study for comparative analysis of an unrelated viral protein with an alternative immunomodulating mechanism of action. Survival, serum ALT levels and histopathology were assessed 24 h and 10 days post-LIRI.

**Results:**

Serp-2 treatment significantly improved survival to 85.7% percent versus saline-treated wildtype mice (*p* = 0.0135), while M-T7 treatment did not significantly improve survival (*p* = 0.2584). Liver viability was preserved by Serp-2 treatment with a significant reduction in serum ALT levels (*p* = 0.0343) and infarct scar thickness (*p* = 0.0016), but with no significant improvement with M-T7 treatment. Suzuki scoring by pathologists blinded with respect to treatment group indicated that Serp-2 significantly reduced hepatocyte necrosis (*p =* 0.0057) and improved overall pathology score (*p* = 0.0046) compared to saline. Immunohistochemistry revealed that Serp-2 treatment reduced macrophage infiltration into the infarcted liver tissue (*p* = 0.0197).

**Conclusions:**

Treatment with Serp-2, a virus-derived inflammasome and apoptotic pathway inhibitor, improves survival after liver ischemia-reperfusion injury in mouse models. Treatment with a cross-class immune modulator provides a promising new approach designed to reduce ischemia-reperfusion injury, improving survival and reducing chronic transplant damage.

**Electronic supplementary material:**

The online version of this article (10.1186/s12950-019-0215-1) contains supplementary material, which is available to authorized users.

## Introduction

Ischemia-reperfusion injury (IRI) is a two-step process characterized by an initial transient blockade of blood flow and oxygen delivery. With IRI, there is an initial, sub-lethal damage followed by restoration of blood flow and a paradoxical acceleration of injury. Induction of liver ischemia-reperfusion injury (LIRI) is inevitable with transplantation surgery, occurring during organ resection, harvest, and graft implant, and can also occur with trauma and hemorrhagic shock [1]. IRI is a primary cause of early graft failure after transplant and can lead to a higher incidence of early acute and also long term chronic rejection. This ongoing injury to transplanted organs contributes to significant graft loss after the first year post transplant, creating the need for repeat transplantation and is one cause for the acute shortages of donor organs available for transplantation [1].

As ischemia-reperfusion injury is seen often during transplantation, attention has been centered on identifying methods to reduce IRI during and post-transplant (e.g., liver, lung, heart, and kidney transplants). For liver grafts, the post-transplantation standard of care commonly involves targeting IL-2 with either monoclonal antibodies (e.g., Basiliximab) [2], mycophenolic acid [3] or FK506 [4] (frequently as a cocktail) in an effort to prevent T cell proliferation and activity. Other approaches include inhibition of the mammalian target of rapamycin (mTOR) with drugs such as rapamycin and Everolimus [5], or generalized immune suppression with cyclosporine A or steroids [6]. While these treatments have significantly improved outcomes following liver transplantation in the past three decades, many adverse effects persist. Most notably, transplant recipients are at increased risk for malignancy [7], viremia [8, 9], bone loss [10], new-onset diabetes [11] and cardiovascular disease [12], many of which are associated with post-transplant immune suppression. Experimental approaches to reduce post-ischemic injury while also avoiding these adverse effects have included activation or inactivation of specific signaling pathways by genetic or small molecular perturbation [13–15], and pre- or post-treatment with a variety of small molecules [16–18]. Despite experimental advances, movement towards the clinic has been slow and there is a substantial need for steroid-sparing, immune modulatory treatments designed to prevent tissue loss after IRI.

Viruses have evolved advanced and highly potent immune-modulating strategies over millions of years of co-evolution with mammalian hosts. A key example of one such virus is Myxomavirus, a leporipoxvirus and the causative agent of a lethal infection, myxomatosis, in the European rabbit (*Oryctolagus cuniculus*). Interestingly, due to incompletely understood mechanisms, Myxomavirus is host-restricted to *O. cuniculus* and is not pathogenic in other rabbit species and in humans [19].

Myxomavirus has evolved to a highly effective pathogen in rabbits through development of potent immune modulating proteins deployed to subvert, suppress and overwhelm the host immune response. We have previously demonstrated therapeutic benefit through delivery of these immune modulators as either recombinant, purified proteins or a coding sequence DNA in Adeno-associated viral vectors (AAV) in animal model studies of disease. For example, the Myxomavirus protein M-T7 is a chemokine-GAG interaction inhibiting protein that reduces renal transplant rejection in both rats [20] and mice [21], and decreases vascular balloon injury in rabbits and rats [22]. In other work, we have demonstrated that treatment with Serp-1, a member of the serpin superfamily of proteins, as well as peptides derived from the Serp-1 reactive center loop (RCL), reduce severity and prolong survival in a lethal, herpesvirus-induced model of large vessel vasculitis [23–25]. These and other examples demonstrate that immune modulatory proteins employed by Myxomavirus for anti-immunological evasion are attractive proteins for repurposing as new therapeutic approaches.

Serp-2 is a second Myxomavirus-derived serpin that is a critical virulence factor for Myxomavirus. Viruses deficient in Serp-2 cause robustly attenuated infections with substantial increases in virus-limiting inflammation [26]. Early molecular work on Serp-2 has demonstrated cross-class inhibitor activity for caspase-1 in the inflammasome signaling pathway, as well as caspases 8 and 10 and granzyme B in the apoptosis pathway [27–29]. Thus, by inhibiting both inflammasome and apoptotic signaling, Serp-2 enables Myxomavirus to suppress inflammation and avoid immune clearance.

In prior work, we tested Serp-2 treatment as an immune modulatory, anti-inflammatory protein therapeutic to reduce disease pathology in mouse models. A single administration of Serp-2 treatment significantly reduced aortic aneurysm formation and plaque growth in an aortic angioplasty model in Apolipoprotein E-deficient (ApoE^−/−^) mice over a period of 4 weeks [30]. In other work, Serp-2 potently reduced plaque growth and inflammation in two separate models: a rat model of iliofemoral balloon angioplasty injury, as well as aortic allograft transplant of plasminogen activator inhibitor 1-deficient (PAI-1^−/−^) or ApoE^−/−^ aortas into Balb/C recipient mice [31]. Serp-2 lost activity in granzyme B/ApoE double knock-out aortic allograft transplants. Interestingly, in a carotid cuff injury model in ApoE^−/−^ mice, Serp-2 displayed systemic effects against plaque growth at the aortic root, a site distal to the acute cuff injury [31]. Thus, Serp-2 has been demonstrated as an effective and potent systemic, cross-class immune modulator against tissue injury in a variety of inflammatory in vivo models.

This short report extends prior studies with Serp-2 as a virus-derived, therapeutic immune modulator to an analysis of the potential for treatment with Serp-2 in a mouse model for LIRI. Progression of LIRI has been attributed to a variety of cellular mechanisms. Among the proposed mechanisms, perturbation of the apoptotic and inflammasome signaling cascades has demonstrated efficacy in in vivo models [14, 32–38]. On this basis, we hypothesized that the apoptosis and inflammasome inhibitory functions of Serp-2 would reduce pathology in liver ischemia-reperfusion injury. Here, we investigated LIRI as a controlled, outcomes-focused (i.e., survival) model for testing further applicability of Serp-2 as a therapeutic protein.

## Methods

### Mouse liver ischemia reperfusion injury (LIRI)

All animal protocols were approved by the University of Florida Institutional Animal Care and Use Committee (IACUC) and conform to national guidelines. All animals received care in compliance with the Principles of Laboratory Animal Care and National standards. A total of 35 mice had liver ischemia reperfusion injury (LIRI) with 70% surgical occlusion of the hepatic blood supply; five mice had a sham operation. From the occlusion groups, 22 mice had follow-up for 10 days (Saline, *N* = 10; Serp-2, *N* = 8; M-T7, N = 8) and 12 mice had follow-up at 24 h (Sham, *N* = 5; LIRI Saline, *N* = 6; LIRI Serp-2, *N* = 3; LIRI M-T7, N = 3). A detailed description of mouse numbers and treatments used in this study is given in Table 1. Serp-2 or M-T7 (100 ng/g) in 100 μL saline was administered by intraperitoneal bolus through a 30-gauge needle, given 30 min prior to LIRI and then on alternate days for a total of 5 doses. Control mice received 100 μL saline in the same regimen. All mice surviving to 10 days were euthanized.

**Table 1:** Numbers of mice

	Treatment	Follow-up	# C57BL6/J Mice
Sham	N/A	24 h	5
70% Ischemia-Reperfusion	Saline	24 h	6
	Saline	10 days	7
	Serp-2	24 h	3
	Serp-2	10 days	8^a^
	M-T7	24 h	3
	M-T7	10 days	8

^a^One of the original eight mice in this group was censored from analysis due to post-surgical complications

Warm, segmental ischemia to the left and middle hepatic lobes was performed as previously described [39]. Briefly, mice were anesthetized with a ketamine/xylazine mixture. Buprenorphine was given subcutaneously (SC) immediately prior to surgery and postoperatively as an analgesic. After shaving and washing the abdominal area with a three stage betadine soap/alcohol/betadine topical wash, an incision was made using sterile technique from the xiphoid process to the symphysis pubis and the portal vein was exposed. An atraumatic clip was used to interrupt the artery/portal venous blood supply to the left and middle liver lobes (i.e., only the left and middle hepatic artery and portal vein are occluded by the clip to achieve 70% occlusion, while the right branch of portal vein and hepatic artery are patent providing normal blood flow). No intestinal ischemia was seen with this model because the right branch of hepatic blood flow remains open. Wet gauze was used to cover the incision during IR injury. Blanching of the left and middle lobes was observed as confirmation of ischemia. After 90 min of ischemia, the clamp was removed, to allow reperfusion, as confirmed by return of blood flow and return of color. Saline (200–300 μL) was injected subcutaneously as a resuscitation bolus at a site remote from the surgical incision on the dorsum of the mouse. The inner muscle and connective tissue were then closed with absorbent suture (4–0 Coated VICRYL Polyglactin 910 Absorbable Suture) and dermal layers closed with sterile nylon suture. Sutures were removed at 7–10 days post-surgery.

### Protein expression and purification

Serp-2 was His-tagged (His10) at the amino-terminus, expressed from a vaccinia/T7 vector in HeLa cells and purified as previously described [31]. M-T7 was expressed from stabilized CHO cells as previously described [22, 40]. Expressed proteins were immobilized for purification by metal affinity using His-Bind resin (Novagen/Merck Millipore, Burlington, MA, USA). Eluted proteins were found to be > 90% pure by 12% SDS-PAGE following by silver staining and immunoblotting.

### Serum alanine aminotransferase (ALT) measurement

Mice were euthanized at 24 h post-procedure and blood was collected by cardiac puncture and allowed to clot prior to centrifugation and storage of supernatant sera at − 80 °C until measurement. ALT was measured using a kinetic, colorimetric diagnostic assay (#A7526, Pointe Scientific, Canton, MI, USA). Briefly, 10 μL of serum was measured in a total reaction volume of 100 μL by the ALT-catalyzed transfer of the amino group from L-alanine to α-ketoglutarate to form pyruvate and L-glutamate and subsequent reduction of pyruvate and oxidation of NADH to NAD by lactate dehydrogenase. The biochemical reaction was monitored by reduction of absorbance at 340 nm on a Molecular Devices M5e multi-mode plate reader at 37 °C every minute for 5 min in UV-transparent 96-well plates. The ΔOD340*min^− 1^ was converted to IU/L using a standard conversion equation according to the manufacturer.

### Histologic and morphometric analysis

At follow up, 24 h or 10 days, mice were euthanized, and the liver harvested. For in-depth histological analysis we used tissues from mice euthanized at 24 h (Sham, *N* = 5; LIRI Saline, *N* = 6; LIRI Serp-2, *N* = 3; LIRI M-T7, N = 3). Tissues were cut into 0.5 cm^3^ sections for histological analysis. Liver sections were fixed in neutral buffered formalin, paraffin embedded, cut into 4 μm cross sections, and stained with hematoxylin and eosin or Periodic Acid-Schiff. IRI infarct scar area and thickness at 24 h follow up as well as invading mononuclear cell counts were measured by morphometric analysis using an Olympus DP71 camera attached to an BX51 microscope (Olympus America Inc., Center Valley, PA, USA) and quantified using Image Pro 6.0 (MediaCybernetics Inc., Bethesda, MD, USA). Pathophysiologic histologic changes for LIRI were evaluated by pathologists blinded to the treatment given to each mouse based on the Suzuki scoring criteria [17].

### Immunohistochemistry

FFPE sections were rehydrated through graded alcohol and epitopes were retrieved by overnight incubation in sodium citrate buffer at 60 °C. Sections were quenched with 3% hydrogen peroxide in PBS for 15 min at room temperature then blocked for 1 h with 5% BSA in TBST at room temperature. Sections were probed overnight with rabbit polyclonal to F4/80 (1:200 ab75476; Abcam, Cambridge, MA, USA) followed by secondary goat-anti-rabbit HRP-conjugated antibody (ab97051, Abcam) at a dilution of 1:500. Immunoreactivity was revealed using ImmPACT DAB (Vector Labs, Burlingame, CA, USA) and sections were counterstained with hematoxylin, dehydrated and mounted with Cytoseal XYL (Thermo Scientific, Waltham, MA, USA). Positively stained cells were counted in three high-power field areas (100× oil immersion) in each liver cross section.

### Immunoblotting

Frozen liver tissues were homogenized in RIPA lysis buffer (Boston BioProducts) containing a 1× protease inhibitor cocktail (Bimake) using a blade homogenizer. Homogenized samples were rotated at 4 °C for 2 h, pelleted at 15,000 *g* for 20 min at 4 °C and supernatant transferred to new tubes. Protein isolates were quantified by BCA assay (Thermo Scientific), normalized with RIPA buffer and boiled with 1× final concentration reducing Laemmli buffer (Alfa Aesar) at 95 °C for 15 min. Proteins (35 μg/sample) were resolved on a 15% SDS-PAGE, transferred to a 0.2 μm pore PVDF membrane, blocked with 5% non-fat dry milk in 0.1% TBS-Tween 20 and probed with primary antibodies against actin (1:600, Rabbit polyclonal, Sigma Aldrich #A2066), cleaved caspase-3 (1:1,000, Rabbit monoclonal, Cell Signaling # 9664S), caspase-8 (1:1,000, Mouse monoclonal, Proteintech #66093-1-IG) or caspase-1 (1:1,000, Mouse monoclonal, Adipogen #AG-20B-0042-C100) overnight in blocking buffer at 4 °C with rocking. Secondary HRP-conjugated antibodies against mouse (Jackson ImmunoResearch #115-035-062) or rabbit (Jackson ImmunoResearch #111-035-144) were incubated at room temperature for 2 h in blocking buffer with shaking. Proteins were revealed with Amersham ECL Start (actin; GE #RPN3243) or ECL Prime (caspases 1, 3 and 8; GE #RPN2236) on a GE LAS4000 imager on the high resolution setting in 10 s increment developments until desired image quality was achieved. Densitometry analysis of cleaved caspase bands normalized to actin was performed in Image Studio Lite v5.2.5 (Li-Cor Biosciences) using the Top/Bottom averaging background correction method with a border width of 3.

### Statistical analysis

Statistical analysis was performed using GraphPad Prism version 8 (GraphPad, La Jolla, CA, USA). Mean IR injury area and cell count from three sections per infarcted liver were analyzed by analysis of variance (ANOVA) with Fishers PLSD (Protected Least Significant Difference) and unpaired, two-tailed Student’s T-test secondary analysis (*p* < 0.05 considered significant). Cumulative survival was performed using the Kaplan-Meier survival analysis with the Mantel-Cox statistical post-hoc test.

## Results

### Serp-2 treatment reduces acute injury and improves survival in the mouse LIRI model

**Fig. 1:**
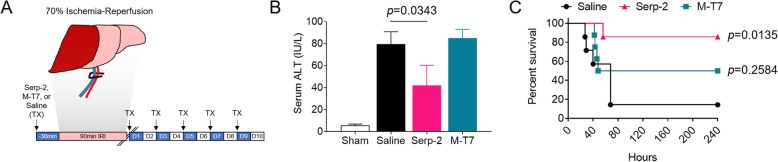
Serp-2 treatment improves survival following liver ischemia-reperfusion injury. (**a**) Experimental outline. Mice were treated with Serp-2, M-T7 or saline (treatment; TX) 30 min prior to induction of 70% ischemia-reperfusion maintained for 90 min and were treated with Serp-2, M-T7 or saline on alternating days for 10 days. (**b**) ALT levels in the serum of sham-operated mice or mice treated with saline, Serp-2 or M-T7 mice with ischemia-reperfusion injury at 24 h post-procedure. Statistics calculated by one-way ANOVA with Fisher’s PLSD post-hoc analysis (*N* = 2–6 mice per group). (**c**) Mice treated with Serp-2 (magenta triangles) had significantly improved survival outcomes compared with mice treated with saline, while mice treated with M-T7 (teal squares) did not show improved survival. Kaplan Meier curve statistics calculated by Log-rank (Mantel-Cox) test (*N* = 7–8 mice per group)

The effects of Serp-2 or M-T7 treatment on LIRI were assessed after warm 70% occlusion of hepatic blood flow for 90 min (Fig. 1A). Serp-2 treatment at a dose of 100 ng/g delivered i.p. immediately prior to induction of LIRI significantly reduced serum levels of alanine aminotransferase (ALT), a clinical diagnostic marker for liver injury, at 24 h (*p* = 0.0343). In comparison, M-T7, a virus-derived chemokine inhibitor that has unrelated immune inhibitory functions, when given at the same dose did not reduce ALT levels when compared to saline (Fig. 1B). Serp-2 significantly reduced mortality with 6 out of 7 C57BL6/J wildtype (WT) mice surviving to 10 days with only one early loss at 56 h (*p* = 0.0135) when compared to saline treated WT mice, in which 6 out of 7 mice died by 10 days (Fig. 1C). M-T7 showed partial effectiveness with 4 of 7 mice survival to 10 days, but this increased survival was not significant (Fig. 1C). Thus, Serp-2 treatment alone was sufficient to prolong short-term survival in mice post-LIRI.

### Serp-2 treatment reduces post-ischemic liver injury

**Fig. 2:**
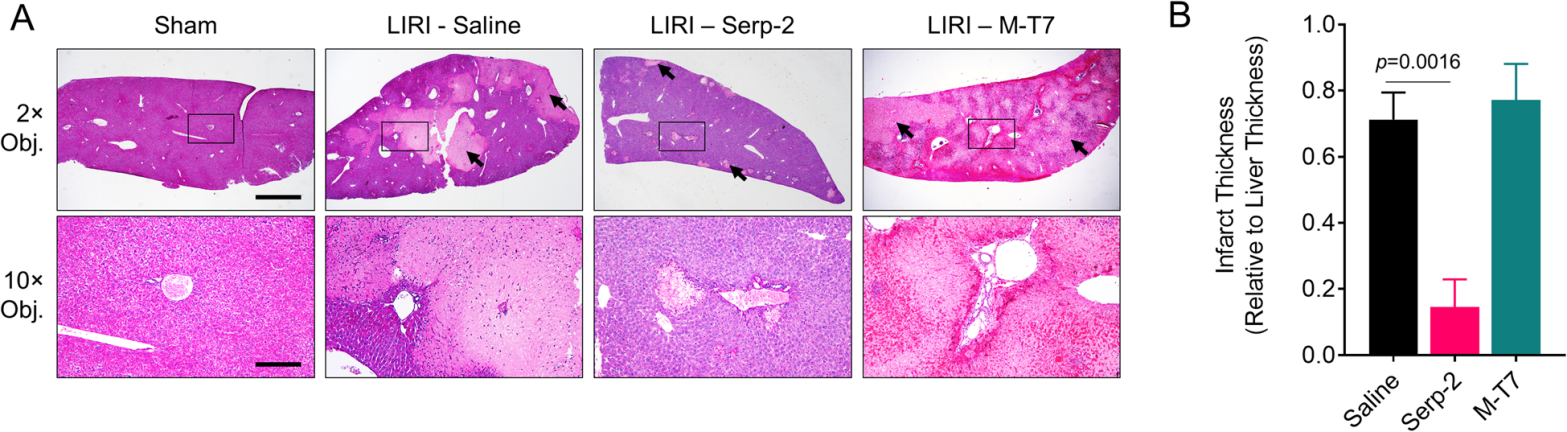
Serp-2 preserves tissue viability after ischemia-reperfusion injury. (**a**) Representative images of sham operated or LIRI-induced mice treated with saline, Serp-2 or M-T7 with 2× or 10× objectives (20× and 100× magnification, respectively). 10× objective image regions are indicated by black boxes in the 2× objective images. Scale bars represent 1000 μm (2× obj.) and 200 μm (10× obj.). Infarcted tissue is indicated with black arrows. (**b**) Relative measure of infarct thickness in livers of LIRI-induced mice treated with saline, Serp-2 or M-T7. Statistics calculated by one-way ANOVA with Fisher’s PLSD post-hoc test (N = 2–5 mice per group)

We next investigated the effect of Serp-2 or M-T7 treatment on maintenance of liver viability after ischemia-reperfusion. When compared to sham-operated mice, histopathology clearly demonstrates a significant increase in hepatocyte necrosis in saline-treated mice. This tissue necrosis was ameliorated by treatment with Serp-2, but not by M-T7 (Fig. 2A). Regions of the liver affected by IRI that developed evidence for infarction and scarring had significantly reduced infarct areas after treatment with Serp-2, but not with M-T7 when compared to saline at 24 h follow up (Fig. 2B; *p* = 0.0016). At this point in our study, we determined that M-T7 was not effective in this model and thus focused on the mechanism and therapeutic benefit of Serp-2.

Independent histopathological analysis by pathologists blinded to treatments, indicated that numerous indicators of liver viability were improved with Serp-2 treatment (Table 2). Compared to saline controls, Serp-2 treatment significantly reduced liver necrosis (*p* = 0.0057) with a strong trend towards significance in reducing hepatocyte vacuolization (*p* = 0.0631) and a modest reduction in congestion (*p* = 0.5128). Aggregate overall pathology score indicated significant improvement with Serp-2 treatment (*p* = 0.0046). Protection against worsened injury by Serp-2 treatment was not due to prevention of caspase-1, − 3 or − 8 cleavage (Additional file 1: Figure S1).

**Table 2:** Suzuki scores of mice treated with saline or Serp-2

Category	Saline	Serp-2	*P*-value^a^
Congestion	2.000 ± 0.2532	1.700 ± 0.3958	0.5128
Vacuolization	2.692 ± 0.2083	2.000 ± 0.2981	0.0631
Necrosis	2.615 ± 0.2895	1.500 ± 0.1667	**0.0057**
Overall Pathology	2.437 ± 0.1157	1.734 ± 0.2035	**0.0046**

^a^*P*-values were calculated by unpaired, two-tailed T-test. Significance (*p*<0.05) is indicated by bolded text

### Serp-2 reduces early inflammatory infiltration to infarcted post-ischemic liver tissue

**Fig. 3:**
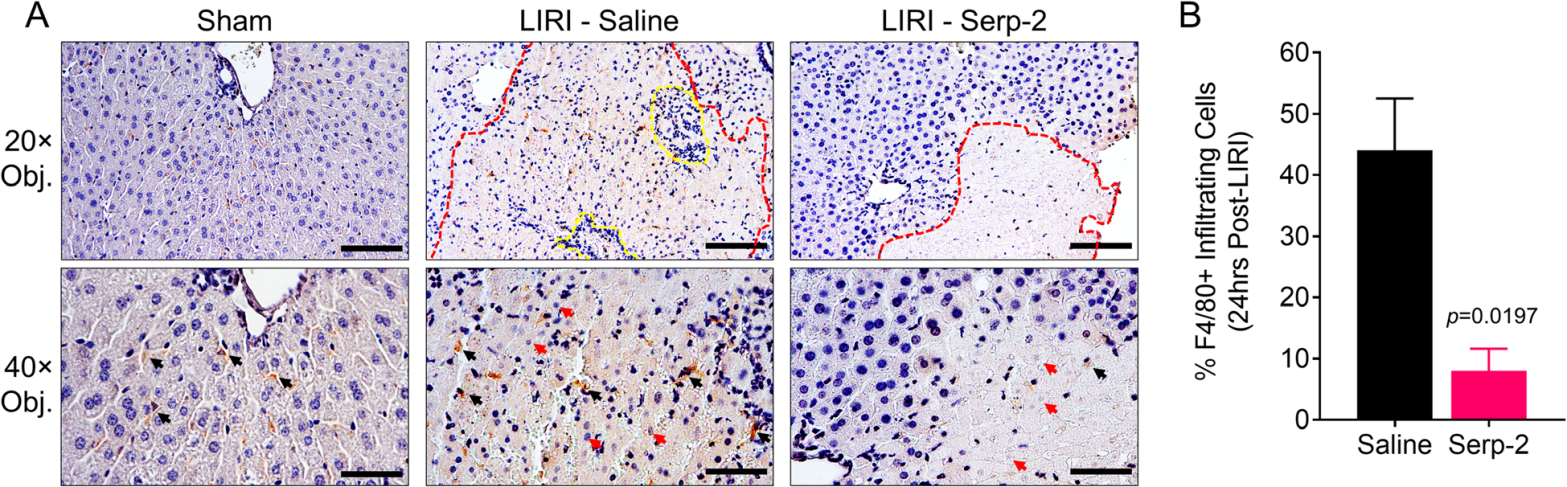
Serp-2 treatment suppressed macrophage infiltration into post-ischemic infarct tissue. (**a**) Representative fields at 20× and 40× magnification of a sham-operated liver and from post-ischemic infarcted tissue in livers from saline or Serp-2 treated mice stained with an antibody against F4/80 and counterstained with hematoxylin. Dashed red region indicates infarcted tissue as determined by necrotic hepatocytes. Dashed yellow region indicates inflammatory cell infiltrates. Black arrows indicate F4/80-positive cells. Red arrows indicate necrotic hepatocytes. Scale bars are 100 μm (20×) and 50 μm (40×). (**b**) Percentages of F4/80-positive infiltrating cells per high-power field in the infarcted tissue of livers from saline or Serp-2 treated mice. Bars represent mean and standard error. Statistics calculated by Student’s T-test

We initially observed a reduction in the number of non-specific inflammatory cells in Serp-2 treated livers by H&E staining (small, dense nuclei). Macrophage-driven inflammation has been previously reported to drive liver ischemia-reperfusion injury in an inflammasome-dependent manner [36]. Monocyte/macrophage infiltration into post-transplant livers is also associated with worsened outcomes [41]. We thus investigated whether Serp-2 suppressed invading macrophage counts after LIRI. Immunohistochemical staining for the F4/80 pan-macrophage antigen revealed a marked reduction in the number of macrophages detected in the infarct scar zone of post-ischemic livers at 24 h (Fig. 3; *p* = 0.0197).

## Discussion

Despite advances in surgical procedure techniques and post-transplant care and immunosuppression, IRI remains a primary cause for early graft loss after liver transplantation [1]. While questions remain as to the exact mechanism of graft loss caused by LIRI, many groups have shown a crucial role for apoptotic [42, 43] and also inflammasome pathway activation and signaling [14, 34, 36, 38]. Study of these pathways has led to substantially improved treatments in other sterile diseases, and thus attention has now been focused on developing similar treatments in LIRI.

Here, we investigate the potential for Serp-2, a Myxomavirus-derived serine proteinase inhibitor (serpin) with known cross-class inhibition of caspase-1 in the inflammasome signaling cascade and caspases 8 and 10 and granzyme B in the apoptosis signaling cascade, to ameliorate LIRI severity in mice. We have demonstrated here that while saline-treated mice die early after LIRI, repeated injections of Serp-2 (every alternate day) at a dose of 100 ng/g, in the absence of other immune suppression treatments, significantly prolonged survival for up to 10 days. Ten days was the endpoint of our study (Fig. 1C). Protection against ischemia-reperfusion injury was likely initiated early and not due to accelerated healing as ALT, an indicator of acute liver injury, was reduced by Serp-2 at 24 h (Fig. 1B). The improved survival and reduced damage were specific to the function of Serp-2. Similar same-dose treatments with M-T7, an unrelated Myxomavirus-derived immune modulating protein, did not provide any survival advantage, reduction in acute injury markers nor preservation of tissue viability (Figs. 1 and 2). We also note that not only do mice treated with Serp-2 survive, but their livers display significant reductions in necrosis and overall pathology when compared to saline-treated mice (Fig. 2 and Table 2). In investigating a potential physiologic mechanism for Serp-2-dependent survival advantage in this model, we note that in addition to reduced infarct scarring of the liver, areas of infarct had smaller inflammatory cell infiltrates, identified as macrophages by immunohistochemistry (Fig. 3). While we cannot specifically identify whether the macrophage infiltrates in the infarcted region are so-called “cavity” macrophages [44] or tissue-resident Kupffer cells, we note that the survival outcomes from Serp-2 treatment combined with the reduction of invasive monocyte-lineage inflammatory cells agrees with prior clinical studies [41].

Ischemia-reperfusion injury creates a complex physiological state in the liver, involving hypoxia, reactive oxygen and nitrogen species formation, multiple forms of cell death and subsequent damage associate molecular pattern (DAMP) release, all of which initiate a feed-forward cascade of damage [45]. Indeed, the mechanism of IRI in the liver remains a topic of active debate, particular with respect to the role of apoptosis, necrosis and other forms of cell death in propagating post-ischemic tissue damage [37, 42, 46, 47]. Accordingly, caspase cleavage has been reported even in the presence of a number of other small molecule and biologic treatments shown to preserve liver viability during warm IR procedures [48–51]. Here, we found profound protection against ischemia-reperfusion injury by treatment with Serp-2, despite still observing caspase-1, − 3 and − 8 activation at 24 h follow-up (Additional file 1: Figure S1). Our data therefore agree with the principle that activation of apoptotic and inflammatory caspases is not by itself a direct nor the sole indicator of tissue viability or injury following ischemia-reperfusion, and that histopathology or functional readouts (e.g., circulating markers of injury such as ALT) are preferred for assessing the effect of protection in this model [37]. It should also be noted that Serp-2 may alter circulating levels of proteases rather than tissue levels and that the tissue isolates will represent a composite of multiple cell types that may respond heterogeneously to the Serp-2 mediated protective functions. Thus, the precise physiological mechanism and the precise cell targets for Serp-2 protection against ischemia-reperfusion injury in the liver, as described in this brief report, remains to be elucidated. Further work will focus on potential extracellular effects of Serp-2, such as inhibition of extracellular and circulating active caspases released from dying cells, a recently reported mediator of inflammation amplification [52–54], or on potential therapeutic benefit of Serp-2-derived metabolites and peptides, such as we have described for Serp-1, a related serpin from Myxomavirus [24, 25].

Myxomavirus-derived proteins as therapeutic agents have proven highly effective in a wide variety of animal models [20, 21, 55, 56]. Of importance, the evolution of these proteins within poxvirus vectors has led these proteins to exhibit very low immunogenicity. Indeed, Serp-1 was proven safe and effective in a Phase IIa clinical trial in humans with acute unstable coronary syndrome [57]. Serp-1 dose-dependently reduced markers of heart damage with a Major Adverse Cardiac Event (MACE) score of zero and without induction of neutralizing antibody. The safety and cross-species efficacy of Myxomavirus-derived proteins, and in this study with Serp-2, highlights the potential for developing these agents for treatment of inflammatory diseases. The substantial and significant Serp-2 mediated therapeutic benefit post-LIRI as demonstrated in this study indicates the potential for Serp-2 treatment in liver transplantation. Further study is warranted for testing Serp-2 and other Myxomaviral proteins in preserving engrafted tissues.

## Additional file

Additional file 1: **Figure S1.** Serp-2 mediated protection against LIRI at 24 hours does not prevent cleavage of caspases 1, 3 and 8. (**a**) Immunoblot analysis of 2 mice each from sham surgery or from 90 minutes liver ischemia-reperfusion injury at 24 hours follow-up probed for antibodies against cleaved caspase-3 (p19), full length/cleaved caspase-8 (p45/p18) and full length/cleaved caspase-1 (p45/p20) with actin as a loading control. (**b**) Densitometry of cleaved bands for caspases-1, -3 and -8 normalized to actin. Statistics performed by 2-Way ANOVA with Fisher’s LSD. (TIF 361 kb)

## Data Availability

All data generated or analyzed during this study are included in this published article.

## Abbreviations

AAV: Adeno-associated viral vectors
ALT: Alanine aminotransferase
ApoE: Apolipoprotein E
H&E: hematoxylin and eosin
IP: intraperitoneal
IRI: Ischemia-reperfusion injury
LIRI: Liver ischemia-reperfusion injury
mTOR: mammalian target of rapamycin
PAI-1: plasminogen activator inhibitor-1
SC: subcutaneous
serpin: serine proteinase inhibitor
WT: wildtype
