# Supplementary figures and images for: Serp-2, a virus-derived apoptosis and inflammasome inhibitor, attenuates liver ischemia-reperfusion injury in mice
Source: J Inflamm (Lond). 2019 May 29;16:12. doi: 10.1186/s12950-019-0215-1 (PMC6542089; doi:10.1186/s12950-019-0215-1)

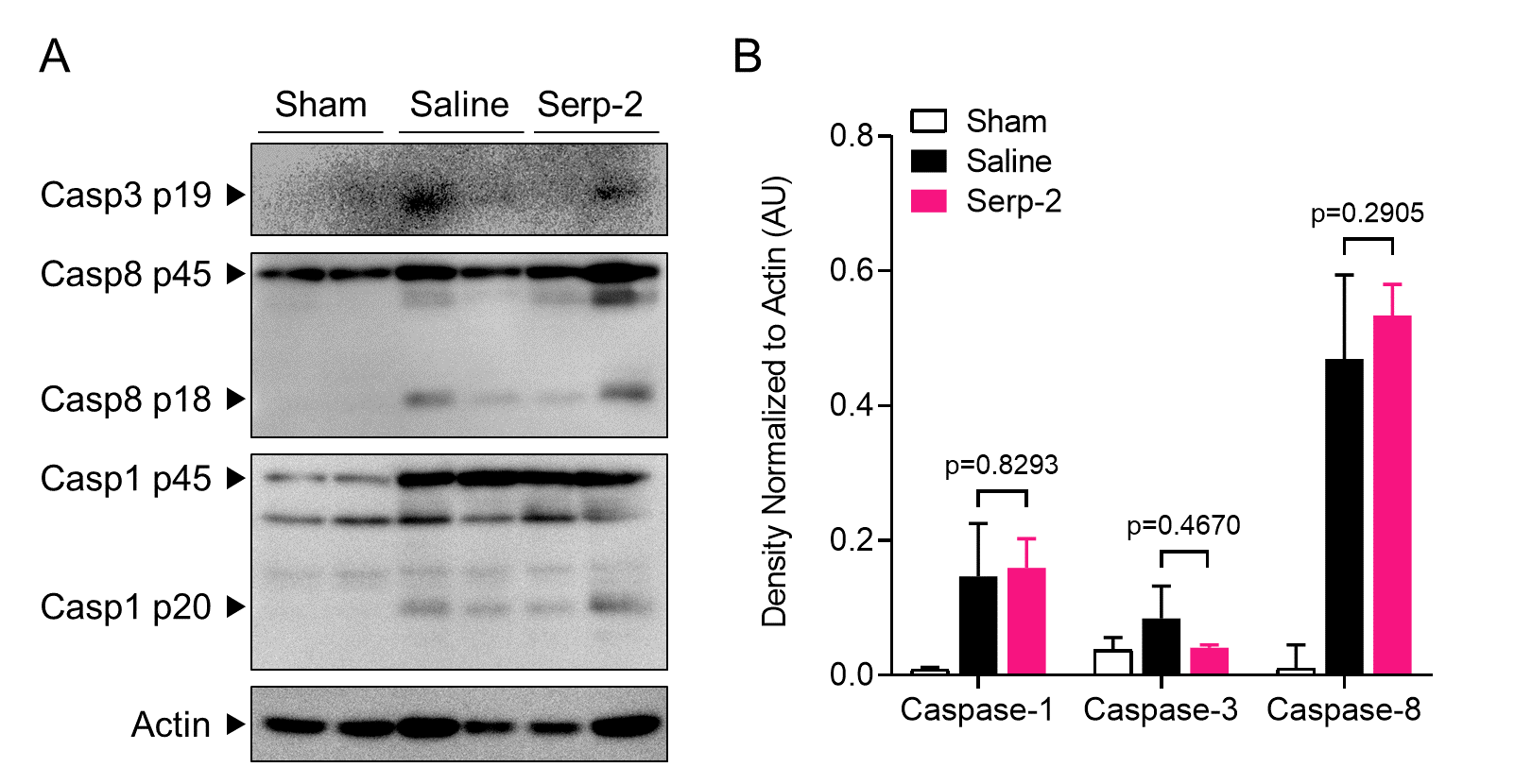

Supplement: Supplementary file 1 — Figure S1. Serp-2 mediated protection against LIRI at 24 hours does not prevent cleavage of caspases 1, 3 and 8. (a) Immunoblot analysis of 2 mice each from sham surgery or from 90 minutes liver ischemia-reperfusion injury at 24 hours follow-up probed for antibodies against cleaved caspase-3 (p19), full length/cleaved caspase-8 (p45/p18) and full length/cleaved caspase-1 (p45/p20) with actin as a loading control. (b) Densitometry of cleaved bands for caspases-1, -3 and -8 normalized to actin. Statistics performed by 2-Way ANOVA with Fisher’s LSD. (TIF 361 kb) [file 12950_2019_215_MOESM1_ESM.tif]
